# Whole-genome sequences of two *Drosophila melanogaster* microbiome symbionts

**DOI:** 10.1128/MRA.00602-23

**Published:** 2023-10-13

**Authors:** Alexander J. Barron, Nichole A. Broderick

**Affiliations:** 1 Department of Biology, Johns Hopkins University, Baltimore, Maryland, USA; The University of Arizona, Tucson, Arizona, USA

**Keywords:** gut microbiome, genome analysis, symbiosis, lactic acid bacteria, acetic acid bacteria, *Drosophila*

## Abstract

*Lactiplantibacillus plantarum* and *Acetobacter tropicalis* are bacterial symbionts commonly isolated from decaying fruits and from the microbiome of *Drosophila melanogaster*. Studies have shown that these organisms interact synergistically, imparting beneficial effects on the host. Here, we report whole-genome sequences of these microbes obtained from long and short reads.

## ANNOUNCEMENT

**Fig 1:**
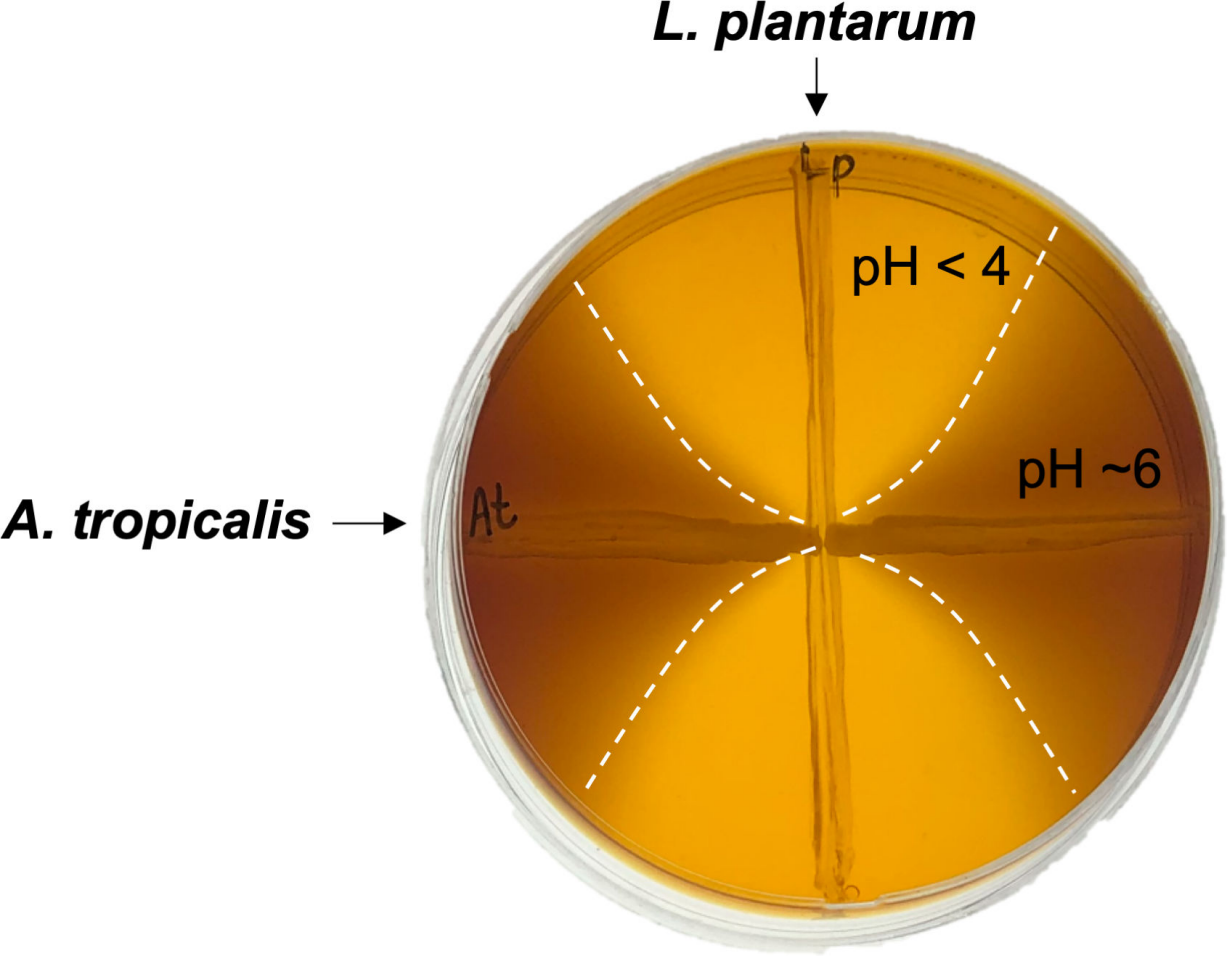
Microbe-microbe interaction assay displaying combinatorial effects of *L. plantarum* and *A. tropicalis* on the pH of culture media. Zones of low pH (<4) surrounding *L. plantarum* are indicated by a yellow coloration and marked with dashed lines.

*Lactiplantibacillus plantarum* NAB1 and *Acetobacter tropicalis* DmCS_006 were isolated from *Drosophila melanogaster* and have been used to model host-microbe interactions (1 – 3). Notably, *L. plantarum* lowers environmental pH, while *A. tropicalis* buffers this shift in pH (Fig. 1). *L. plantarum* and *A. tropicalis* were grown in intersecting lines on mannitol agar containing the pH indicator meta-cresol purple (0.02%) for 3 days at 29°C. pH was inferred from the indicator color.

Prior to DNA extraction, the organisms were cultured in Man, Rogosa, and Sharpe broth (RPI) for 24 h at 30°C. For Illumina sequencing, DNA was extracted from liquid cultures using the PureLink Genomic DNA Mini Kit (Invitrogen). DNA sequencing was done by SeqCenter (Pittsburgh, PA). Libraries were prepared using the Illumina DNA prep kit, and samples were indexed with Integrated DNA Technologies (IDT) 10-bp unique dual indices and sequenced on an Illumina NextSeq 2000. This resulted in 1,628,445 reads (35–151 bp) for *A. tropicalis* and 2,029,581 reads (35–151 bp) for *L. plantarum*. Quality control was performed using FastQC v.1.0.0 using a quality score cutoff of 30. Illumina adapters were removed using Trimmomatic v.0.39 (4).

For Oxford Nanopore sequencing, isolates were sent to SeqCoast (Portsmouth, NH), and new liquid cultures were made for each organism. DNA was extracted using the Monarch High Molecular Weight Extraction kit (NEB). The *L. plantarum* library was prepared using the Oxford Nanopore Technologies SQK-RBK114 rapid barcoding kit and sequenced on the GridION platform with a FLOW-MIN114 Spot-ON flow cell v.R10. *A. tropicalis* was prepared using the Oxford Nanopore Technologies SQK-LSK110 ligation kit and sequenced on the GridION platform using a FLOW-MIN106D Spot-ON flow cell v.R9. This resulted in 442.83 k raw reads (*N*_50_ = 4.73 kb) for *L. plantarum* and 54.88 k reads (*N*_50_ = 14.9 kb) for *A. tropicalis*. Base-calling was done using the MinKNOW super-accurate base-calling model v.22.12.5 with Guppy v.6.4.6. Quality control of reads was performed using the GridION software, with a quality score cutoff of 10. Nanopore adapters were removed using PoreChop v.0.2.4.

Hybrid genomes using Illumina and Nanopore sequences were generated using Unicycler v.0.4.4 (5) as follows: (i) contigs were generated using SPAdes with the default settings (6, 7); (ii) reads were mapped using Bowtie2 and SAMtools (8, 9); and (iii) *L. plantarum* and *A. tropicalis* assemblies were polished with Pilon eight and four times, respectively (10). The *L. plantarum* assembly is 3,500,051-bp long (84× coverage), has a GC content of 44%, and consists of four contigs (*N*_50_ = 3,336,832 bp). The *A. tropicalis* assembly is 3,717,153 bp long (62× coverage) and has a GC content of 55% across five contigs (*N*_50_ = 3,656,948). Genomes were annotated using PGAP v.6.5 (11).

These high-quality genomes will be useful for studying the symbiosis between lactic acid bacteria and acetic acid bacteria and their interactions with hosts.

## Data Availability

*Lactiplantibacillus plantarum* data are under GenBank accession JAUYUV000000000, and raw sequencing reads are under SRA accession numbers SRR25073487 (Illumina) and SRR25073486 (Nanopore). *Acetobacter tropicalis* data are under BioProject accession JAUYUW000000000. Raw sequencing reads are under SRA accession numbers SRR25073980 (Illumina) and SRR25073979 (Nanopore).
